# Using Satellite-Based Spatiotemporal Resolved Air Temperature Exposure to Study the Association between Ambient Air Temperature and Birth Outcomes in Massachusetts

**DOI:** 10.1289/ehp.1308075

**Published:** 2015-04-07

**Authors:** Itai Kloog, Steven J. Melly, Brent A. Coull, Francesco Nordio, Joel D. Schwartz

**Affiliations:** 1 Department of Geography and Environmental Development, Ben-Gurion University of the Negev, Beer Sheva, Israel; 2 Exposure, Epidemiology and Risk Program, Department of Environmental Health, and; 3 Department of Biostatistics, Harvard T.H. Chan School of Public Health, Boston, Massachusetts, USA

## Abstract

**Background:**

Studies looking at air temperature (Ta) and birth outcomes are rare.

**Objectives:**

We investigated the association between birth outcomes and daily Ta during various prenatal exposure periods in Massachusetts (USA) using both traditional Ta stations and modeled addresses.

**Methods:**

We evaluated birth outcomes and average daily Ta during various prenatal exposure periods in Massachusetts (USA) using both traditional Ta stations and modeled address Ta. We used linear and logistic mixed models and accelerated failure time models to estimate associations between Ta and the following outcomes among live births > 22 weeks: term birth weight (≥ 37 weeks), low birth weight (LBW; < 2,500 g at term), gestational age, and preterm delivery (PT; < 37 weeks). Models were adjusted for individual-level socioeconomic status, traffic density, particulate matter ≤ 2.5 μm (PM_2.5_), random intercept for census tract, and mother’s health.

**Results:**

Predicted Ta during multiple time windows before birth was negatively associated with birth weight: Average birth weight was 16.7 g lower (95% CI: –29.7, –3.7) in association with an interquartile range increase (8.4°C) in Ta during the last trimester. Ta over the entire pregnancy was positively associated with PT [odds ratio (OR) = 1.02; 95% CI: 1.00, 1.05] and LBW (OR = 1.04; 95% CI: 0.96, 1.13).

**Conclusions:**

Ta during pregnancy was associated with lower birth weight and shorter gestational age in our study population.

**Citation:**

Kloog I, Melly SJ, Coull BA, Nordio F, Schwartz JD. 2015. Using satellite-based spatiotemporal resolved air temperature exposure to study the association between ambient air temperature and birth outcomes in Massachusetts. Environ Health Perspect 123:1053–1058; http://dx.doi.org/10.1289/ehp.1308075

## Background

The increase in temperatures over the last century and continued increases in emissions of greenhouse gases have focused attention on the effects of increasing heat (Crowley 2000). Relatively few studies have examined associations between average daily ambient air temperature during pregnancy (Ta) and pregnancy outcomes. Most published work has focused on the relationship between preterm delivery (PT) and Ta with variable results. One study reported an increased risk of very low birth weight (LBW) delivery (birth weight < 1,500 g) with colder ambient temperature (Hartig and Catalano 2013). Another study found no association between preterm birth (birth at < 37 weeks completed gestation) and a variety of factors including temperature, humidity, and barometric pressure (Lee et al. 2008). In contrast, two studies have reported that PT was associated with increased temperature and humidity (Basu et al. 2010; Lajinian et al. 1997). A study conducted in Australia reported that weekly temperature was positively associated with preterm birth < 37 weeks and stillbirth < 36 weeks gestation (Strand et al. 2012). Schifano et al. (2013) reported that maximum apparent temperature in the 2 days preceding delivery was associated with PT in Rome, Italy, during the warm season; they used models adjusted for air pollution, socioeconomic status, and mother’s health.

It is important to determine whether ambient temperature indeed affects the length of gestation and birth weight at delivery, because LBW delivery has significant short- and long-term health implications. PT (delivery at < 37 weeks gestation), early-term delivery (delivery at 37–38 weeks gestation), and *in utero* growth restriction (IUGR; delivery at birth weight < 10th percentile for gestational age) also contribute to perinatal morbidity and mortality (Harding and Maritz 2012; McCormick 1985; Moster et al. 2008; Sengupta et al. 2013). Evidence suggests that IUGR birth in particular may have long-term implications for childhood and adult health (Bilbo and Schwarz 2009; Demicheva and Crispi 2014; Gluckman and Hanson 2004; Harding and Maritz 2012; Sarr et al. 2012; Vos et al. 2006). The pathogenesis of preterm, early-term, and IUGR delivery is multifactorial. Inflammation, infection, and immune dysregulation may cause preterm labor and early delivery; abnormalities of placental formation and function may result in preterm, early-term, and IUGR delivery due to placental bleeding, fetal distress, and preeclampsia; and genetic variation and multiple gestation contribute to each of these etiologies (Gonçalves et al. 2002; Han et al. 2011; Leber et al. 2010; Muglia and Katz 2010; Saito et al. 2010; Wong and Grobman 2011). Social stressors have also been studied as causes of preterm, early-term, and IUGR delivery, due to variation in the rate of LBW delivery among different racial, ethnic, and socioeconomic groups (Kuzawa and Thayer 2011; Wadhwa et al. 2011). Environmental stressors such as changes in ambient air temperature may also contribute to these birth outcomes. A recent study by Dadvand et al. (2014) examined the association of term low birth weight with residential proximity to major roads and surface temperature. They showed that living within 200 m of major roads was associated with an increase in term LBW risk [odds ration (OR) = 1.46; 95% confidence interval (CI): 1.05, 2.04]. They also found that surface temperature was associated with an increase in term LBW risk (OR = 1.18; 95% CI: 0.95, 1.45). The conflicting results published to date on relationship of ambient air temperature to preterm and/or LBW delivery may be attributable to variations in temperature measurement and modeling. Air temperature stations have limited spatial coverage, particularly in less urban areas, and airport monitors may not reflect the urban heat island adequately. Because temperature can vary greatly both spatially and temporally, the use of air temperature stations can introduce considerable measurement error (and downward bias in the case of heat islands), reducing their utility for epidemiological studies on the health effects of extreme temperature and climate change. Previous studies examining the association of preterm and LBW delivery and Ta have typically used available monitors in the study area. This introduces exposure error and likely biases the effect estimates downward (Armstrong 1998; Zeger et al. 2000). Furthermore, lack of spatially resolved daily Ta concentration data restricts these studies to populations surrounding monitoring sites, which may not be representative of the population as a whole.

The lack of high-resolution continuous spatiotemporal Ta data resulted in our group developing a method to predict 24-hr mean Ta at a very fine spatial resolution (Kloog et al. 2012a, 2014). Specifically, we developed new methodologies to predict daily Ta, based on land use regression plus a daily calibration of Ta ground measurements and MODIS (Moderate Resolution Imaging Spectroradiometer; http://modis.gsfc.nasa.gov/data/) surface temperature (Ts) over a large area with varying geographical characteristics (covering the entire Northeast and Mid-Atlantic areas of the United States) at a 1 × 1 km spatial resolution. We incorporated land use and meteorological variables to predict daily 24-hr mean Ta for grid cells even when satellite Ts measures were not available. A similar model has previously been developed for PM_2.5_ on the same resolution (Kloog et al. 2012c).

We used our Ta prediction data to study associations between Ta and live birth outcomes among singleton births in Massachusetts during 2000–2008, including term birth weight, LBW (< 2,500 g) among term births, preterm birth (< 37 weeks), and gestational age.

## Methods

### Study Domain and Population

**Figure 1:**
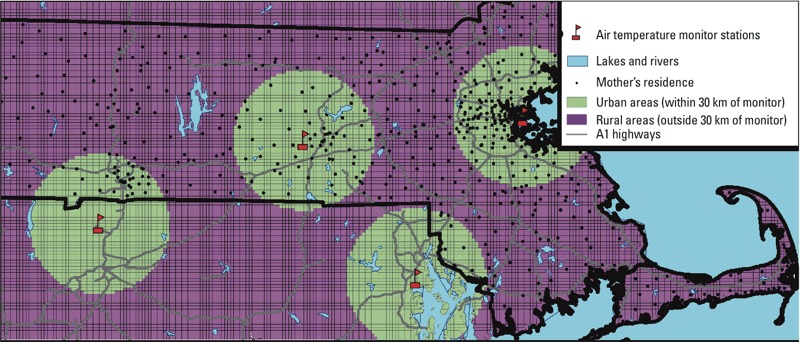
Map of the study area showing the location of a sample subset of mothers [randomly selected with the QGIS tool “random points” (http://www.qgis.org/)], the location of the ground air monitoring stations, and the areas within and outside 30 km of an air temperature station (urban vs. rural areas).

In the analysis we included the entire state of Massachusetts (Figure 1). The study population included all live singleton births > 22 weeks of gestation in Massachusetts from 1 January 2000 through 31 December 2008 (Figure 1). Birth data and the latitude and longitude of each eligible address at birth were provided by the Massachusetts Birth Registry (MBR; http://www.mass.gov/eohhs/gov/departments/dph/programs/admin/dmoa/vitals/). The term birth weight and LBW (< 2,500 g) analyses included 453,658 births ≥ 37 weeks gestational age, and the gestational age and preterm birth (< 37 weeks) analyses included 473,977 births. The study and the use of birth data was approved by the Massachusetts Department of Public Health and the human subjects committee of the Harvard T.H. Chan School of Public Health. Informed consent was not required because we used anonymous administrative data.

### Exposure Data

For exposure data we used three different indicators: predicted 1 × 1 km Ta from our model, ground Ta from the nearest National Climatic Data Center (NCDC; http://www7.ncdc.noaa.gov/CDO/) monitoring stations, and residence-specific cumulative traffic density. We describe each metric in more detail below.

*Predicted air temperature.* Ta exposure data were generated by the previously mentioned Ta prediction model (Kloog et al. 2014). In these prediction models we used mixed models to first calibrate Ts and Ta measurements, regressing Ta measurements against day-specific random intercepts, fixed and random Ts slopes, and several spatial and temporal predictors [Normalized Difference Vegetation Index (NDVI), percent urban and elevation]. Then to make use of the ability of neighboring cells to fill in the cells with missing Ts values, we regressed the Ta predicted from the first mixed-effects model against the mean of the Ta measurements on that day from monitors within 60 km, separately for each grid cell. We used 10-fold of sample cross-validation (CV) to validate our predictions at monitor locations at each step. We randomly divided our data into 90% and 10% splits 10 times. We predicted for the 10% data sets using the model fitted from the remaining 90% of the data. We then reported these computed *R*^2^ values. To test our results for bias, we regressed the measured Ta values against the predicted values in each site on each day. We estimated the model prediction precision by taking the root mean square prediction error (RMSPE). Mean out-of-sample *R*^2^ values for days with and without Ts data were 0.947 and 0.940, respectively, indicating excellent model performance. Mean out-of-sample temporal and spatial *R*^2^ values also were high (0.956 and 0.832, respectively) (Kloog et al. 2014).

**Figure 2:**
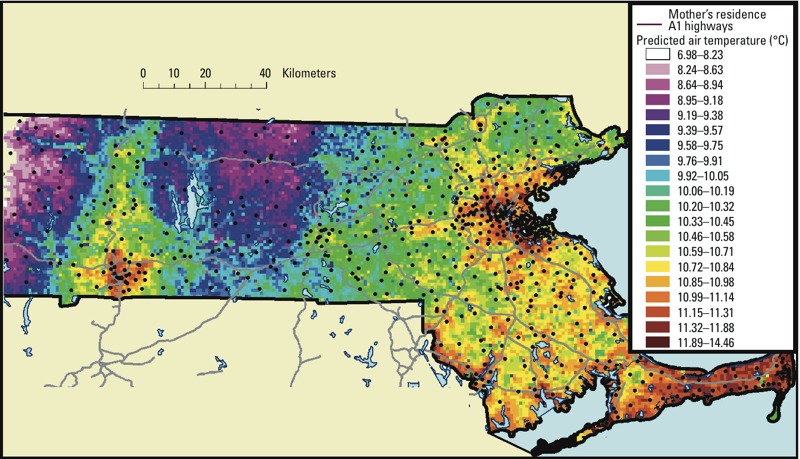
Map of the study area showing the residential location of a subset of mothers over the daily predicted air temperature (°C) 1 × 1 km grid averaged for the entire year of 2005.

To estimate Ta exposure, we linked each mother’s residence at the time of delivery to its corresponding grid cell (Figure 2). Daily Ta exposures were calculated for the day of birth; the day before birth; moving average values for 3 days, 7 days, 14 days, 30 days, the last trimester; and the entire pregnancy.

*PM_2.5_.* Particulate matter ≤ 2.5 μm (PM_2.5_) was estimated on a 1 × 1 km grid from the same MODIS satellite, using daily measures of aerosol optical depth using a similar methodology (daily calibration, land use, and meteorology) as the temperature model. Further details have been published previously (Chudnovsky et al. 2014; Kloog et al. 2012c). Because warm days are often more polluted, PM_2.5_ was included as a covariate with the same time periods used to classify Ta.

*Monitored air temperature.* Daily data for monitored Ta across Massachusetts were obtained from the NCDC. NCDC is a government agency and has been collecting meteorological data for close to a century. Ta is measured at a reference height of 2 m above the ground in most weather stations (NCDC 2010).

*Cumulative traffic density*. Traffic emissions have been associated with birth outcomes in many previous studies (Gryparis et al. 2009; Zeka et al. 2006). Therefore, Massachusetts road data [average daily traffic (ADT)] were obtained from the Massachusetts Department of Transportation (MassDOT; http://www.massdot.state.ma.us/) 2002 Road inventory. These data are based on automatic vehicle counts on major highways, periodic counts on other major roads, and estimated counts for all other roads (Kloog et al. 2012b). Each 200 × 200 m grid was assigned a value for normalized cumulative ADT (CADT) based on average daily traffic on all road segments within 100 m of the center of each grid, where CADT = Σ(ADT × road segment length). Each birth address was assigned the average CADT value for the four grids with center points closest to the address, using bilinear interpolation.

On the basis of previous literature on the potential risk factors associated with low birth weight (Kloog et al. 2012b; Zeka et al. 2006, 2008), we included the following individual and contextual covariates:

Percent of open space. The percent of open space data was obtained from the office of geographic information Commonwealth of Massachusetts, information technology division MassGIS [Massachusetts Office of Geographic Information Executiver Office for Administration and Finance (MassGIS-EOEA) 2006]. The subset of the open space designated for recreation and conservation was intersected with 2000 Census tract boundaries (also downloaded from MassGIS) using ArcGIS^©^ 10.1 (ESRI). The percent of each census tract that was open space was then calculated and assigned to birth addresses belonging to that tract.

Socioeconomic indicators. Socioeconomic data at the individual level were obtained from the Massachusetts birth registry. Information included the mother’s race/ethnicity [classified as Hispanic, non-Hispanic white, African American, Asian, and other (all other ethnic groups)], mother’s years of education, and the Kotelchuck adequacy of prenatal care utilization index (APNCU). The APNCU is based on the number and the time of start of mother’s prenatal visits (Alexander and Kotelchuck 1996) and was recoded into inadequate (< 50% of expected visits used), intermediate (50–79%); appropriate (80–109%), and appropriate plus (≥ 110%) categories. We categorized education of the mother as no high school (< 9 years of educational attainment), some high school (9–12 years of educational attainment); some college (13–15 years); and college or postgraduate (≥ 16 years).

Median income. Data were obtained from the U.S. Census Bureau 1999 median household income (U.S. Census Bureau 2000) for every census tract in the study area, and assigned these to births with an address located within that tract.

Individual-level covariates. Maternal age, parity, gestational age (calculated by the maternal recall of last menstrual period), number of cigarettes smoked per day during and before pregnancy, chronic conditions of mother or conditions of pregnancy (lung disease, pregnancy-induced hypertension, gestational diabetes, and nongestational diabetes, all modeled separately as single variables), previous occurrence of a preterm birth, whether the mother ever had a previous infant weighing ≥ 4,000 g, and sex of infant were all obtained through the Massachusetts Birth Registry (Boston, MA) through the index child’s birth certificate.

### Statistical Methods

To identify factors affecting birth weight, we used linear mixed regression models to estimate associations between both monitor and modeled Ta during different time windows and term birth weight, and logistic mixed regression to estimate associations with preterm birth (< 37 weeks) and LBW (< 2,500 g) (Kloog et al. 2012b; Zeka et al. 2008). Seasonality was controlled using sine and cosine terms with a period of 365.24 days. Both sine and cosine were included to allow the regression to estimate both the amplitude of the seasonal cycle and its phase. A random intercept for census tract was used to capture unmeasured similarities in persons residing in the same neighborhood.

Specifically, we fit the following models:

*BW_ij_ =* (α *+ u_j_*) + β_1_*Ta_i_* + β_2_*PM_i_ +* γ***X****_i_ + e_ij_*(*u_j_*) *~ N*[0,σ*_u_*^2^] and [1]

*Logit* (*PT_ij_/LBW_ij_ = 1|X*) *=* (α *+ u_j_*) *+* β_1_*Ta_i_* + β_2_*PM_i_ +* γ***X****_i_ + e_ij_*(*u_j_*) *~ N*[0, σ*_u_*^2^], [2]

where *BW_ij_*, *PT_ij_,* and *LBW_ij_* represent birth weight, preterm, and LBW, respectively, for the *i*th subject in census tract *j*; α and *u_j_* are the fixed and random (tract-specific) intercepts, respectively; γ***X****_i_* denote the set of variables included in the model, which include predicted ambient air temperature, predicted ambient PM_2.5_, cumulative traffic density, percent of open spaces, age of mother, median income, gestational age, chronic conditions of mother or conditions of pregnancy (lung disease, hypertension, gestational diabetes or nongestational diabetes), parity, previous infant weighing ≥ 4,000 g and sex of infant, sine and cosine (controlling for seasonality), APNCU (as a categorical variable), mother’s race (as a categorical variable), mother’s education (as a categorical variable), and previous preterm occurrences. *e_ij_* is the error term and finally, σ^^2^^*_u_* is the variance of the tract random effects, and *e_jj_* ~ *N*[0, σ^2^].

We estimated associations between Ta during different time windows and gestational age using an accelerated failure time model (AFT).

Such models are a form of survival analysis that model the survival time directly instead of the hazard. Gestational age is used as a continuous outcome in the AFT model. The log-linear form of the AFT model with respect to time (*T*) is given by

log*T_i_* = μ + α_1_*X*_1_*_i_* + α_2_*X*_2_*_i_* + ... + α*_p_X_pi_* + σε*_i_*, [3]

where μ is the intercept, σ is a scale parameter, and ε*_i_* is a random variable, assumed to have a particular distribution. We adopted a gamma distribution for ε*_i_*, which can flexibly model a wide range of distributions for the failure times (births). A two-sided *p*-value < 0.05 was considered statistically significant.

We also ran analyses stratified on subject residence < 30 km or ≥ 30 km of a Ta monitor (as proxy indicators of urban and rural residences, respectively). Statistical analyses were performed in SAS (version 9.3; SAS Institute Inc., Cary, NC) and R (R Core Team 2014). Cases with missing data were excluded from the analysis. An alpha level of 0.05 indicates statistical significance.

## Results

Descriptive statistics are presented in Table 1. Of the 450,407 births included in all births in our analyses, 50% of the births were male, 72% were white, only 8% had maternal age < 20 years for full-term births, and 21% of the mothers had > 15 years of education. Mean (± SD) birth weight was 3,395 ± 502 g among term births and 3,391 ± 511 g among all births. Table 2 contains a summary of the predicted Ta and traffic exposure across all grid cells in the analysis. Table 3 presents the interquartile range (IQR) for each time window used in the analysis. Table 4 presents the results from the regression across all exposure periods tested for both the predicted exposures and monitor exposure analyses. Using our spatially and temporal resolved predicted Ta as exposure resulted in all exposure windows showing decreased birth weights with increased Ta with almost all exposure windows showing statistical significance. We observed a pattern of increasing impact of an IQR change in temperature exposure with increasing averaging time up until the last trimester of gestation average. The effect for the full pregnancy was smaller than that of the last-trimester moving average.

**Table 1:** Characteristics of live births in Massachusetts during the 9-year period 2000–2008 for both the full-term analysis and AFT models.

Characteristic	Term births			All births ≥ 22 weeks		
	Percent of all births (*n*)	Mean birth weight ± SD (g)	Missing (*n*)	Percent of all births (*n*)	Mean birth weight ± SD (g)	Missing (*n*)
Overall	(450,407)	3,395 ± 502	3,251	(462,400)	3,391 ± 511	3,381
Maternal race			0			0
White	72 (323,819)	3,443 ± 496		72 (332,383)	3,440 ± 503
African American	7 (33,775)	3,257 ± 520		8 (34,803)	3,246 ± 540
Hispanic	13 (60,339)	3,297 ± 496		13 (62,029)	3,313 ± 522
Asian	7 (31,491)	3,234 ± 463		7 (32,174)	3,229 ± 473
Other	0.3 (983)	3,347 ± 505		0.3 (1,011)	3,339 ± 527
Maternal education (years)			1,699			1,748
≤ 8	3 (12,718)	3,293 ± 492		3 (13,121)	3,288 ± 501
> 8–12	34 (154,723)	3,319 ± 511		34 (159,307)	3,313 ± 523
13–15	42 (187,792)	3,407 ± 503		42 (192,246)	3,455 ± 491
≥ 15	21 (94,994)	3,457 ± 485		21 (97,726)	3,403 ± 512
Maternal age (years)			1			1
≤ 20	5 (22,900)	3,219 ± 489		5 (23,618)	3,211 ± 505
20–29	33 (146,668)	3,335 ± 518		33 (150,538)	3,330 ± 504
30–34	32 (142,033)	3,427 ± 493		32 (145,720)	3,424 ± 500
35–39	24 (106,542)	3,459 ± 518		24 (109,365)	3,455 ± 512
> 39	7 (32,244)	3,434 ± 518		7 (33,159)	3,431 ± 530
Maternal chronic conditions
Gestational diabetes	3 (15,047)	3,419 ± 633	1,342	3 (15,388)	3,407 ± 562	1,420
Nongestational diabetes	1 (3,128)	3,419 ± 633	1,342	1 (3,219)	3,411 ± 649	1,420
Previous infant ≥ 4,000 g	1 (3,503)	3,936 ± 508	1,342	1 (3,594)	3,937 ± 511	1,420
Hypertension	3 (12,721)	3,258 ± 567	1,342	3 (13,038)	3,253 ± 575	1,420
Lung disease	3 (14,535)	3,295 ± 531	1,342	3 (14,906)	3,287 ± 547	1,420
Previous preterm birth	1 (4,331)	3,080 ± 576	1,342	1 (4,475)	3,070 ± 593	1,420
Gestational age (weeks)	(450,407)	39.0 ± 1.83	0	(462,400)	38.97 ± 1.95	0
APNCU			0			0
1 (inadequate)	9 (40,427)	3,309 ± 507		9 (41,692)	3,304 ± 518
2 (intermediate)	8 (35,519)	3,438 ± 476		8 (36,559)	3,438 ± 477
3 (appropriate)	48 (215,188)	3,465 ± 463		48 (222,638)	3,465 ± 465
4 (appropriate plus)	35 (159,273)	3,312 ± 539		35 (163,511)	3,304 ± 555
Mean household Income (US$)	(453,658)	52,313 ± 21,566	0	(462,400)	52,296 ± 21,573	0
Sex			0			0
Male	50 (226,589)	3,452 ± 511		50 (232,720)	3,447 ± 521
Female	50 (223,818)	3,337 ± 486		50 (229,680)	3,334 ± 494
Parity (number of births)	(450,407)	2 ± 2.7	0	(462,400)	2 ± 2.7	0
Cigarettes per day during pregnancy (smokers)	(450,407)	0.6 ± 2.7	642	(462,400)	0.7 ± 2.7	656
Cigarettes per day before pregnancy (smokers)	(450,407)	1.8 ± 5.1	616	(462,400)	1.8 ± 5.1	631
Cumulative traffic density (average daily traffic counts)	(450,407)	39.4 ± 23.5	0	(462,400)	39.2 ± 23.2	0
Elevation (m)	(450,407)	59.9 ± 68.3	0	(462,400)	60 ± 68.4	0
Percent of open space	(450,407)	12.0 ± 11.1	0	(462,400)	12.0 ± 11.1	0
Season of birth			0			0
Winter	22 (97,982)	3,379 ± 504		24 (108,896)	3,378 ± 514
Spring	26 (117,669)	3,402 ± 502		26 (118,182)	3,399 ± 512
Summer	27 (121,629)	3,399 ± 501		26 (121,917)	3,396 ± 510
Fall	25 (113,127)	3,393 ± 500		25 (113,405)	3,390 ± 508
Abbreviations: AFT, accelerated failure time; APNCU, Adequacy of Prenatal Care Utilization index.						

**Table 2:** Descriptive statistics for daily air temperature, daily PM_2.5_ exposure, and traffic density across mother’s residences (397,698) in Massachusetts between 2000 and 2008.

Covariate	Mean	Median	SD	Minimum	Maximum	IQR	25th percentile	75th percentile	Days of data available
Predicted air temperature (°C)	11.3	11.4	5.6	–12.1	35.49	8.9	6.9	15.8	3,285
Cumulative traffic density (daily traffic × length)	1,309	702	2,076	0	29,000	1,352	258	1,611	3,285
Predicted PM_2.5_ (μg/m^3^)	10.9	9.27	5.9	0.2	56.9	6.8	6.7	13.5	3,285

**Table 3:** IQR (interquartile range) values for each time window used in the study.

Exposure period	IQR (°C)
Day of birth	8.9
One day before birth	8.9
Moving average of 3 days before birth	9.0
Last week (7 days before birth)	9.0
Last 2 weeks (14 days before birth)	9.0
Last month (30 days before birth)	9.1
Last trimester	8.4
Entire pregnancy	2.7

**Table 4:** The adjusted association between a one interquartile range increase in air temperature (°C) and PM_2.5_ and birth weight for full-term births at various exposure periods (*n* = 453,658).

Exposure period	Predicted air temperature (°C)β (95% CI)	Closest monitor temperature (°C)β (95% CI)
Day of birth	–3.6 (–8.1, 0.9)	0.6 (–4.8, 6.0)
One day before birth	–4.4 (–9.6, 0.7)	1.8 (–3.8, 7.5)
Moving average of 3 days before birth	–4.1 (–9.8, 1.5)	3.0 (–2.8, 8.8)
Last week (7 days before birth)	–8.9 (–16.2, –1.5)	1.5 (–4.6, 7.7)
Last 2 weeks (14 days before birth)	–15.5 (–24.2, –6.8)	0.5 (–6.4, 7.3)
Last month (30 days before birth)	–16.6 (–27.4, –5.9)	2.0 (–6.3, 10.2)
Last trimester	–16.7 (–29.7, –3.7)	–8.0 (–20.3, 4.3)
Entire pregnancy	–5.0 (–7.8, –2.3)	2.6 (–17.1, 22.4)
All models adjusted for predicted air temperature, predicted PM_2.5_, cumulative traffic density, percent of open spaces, age of mother, gestational age, chronic conditions of mother or conditions of pregnancy (lung disease, hypertension, gestational diabetes, or nongestational diabetes), parity, previous infant weighing ≥ 4,000 g and sex of infant, sine and cosine (controlling for seasonality), APNCU (as a categorical variable), mother’s race (as a categorical variable), mother’s education (as a categorical variable), and previous preterm occurrences.		

Term birth weights were negatively associated with predicted Ta in almost all exposure time windows (Table 4). In general, the average estimated difference in term birth weight with an 8.4°C (IQR) increment in Ta increased as the averaging time increased up to the last trimester before birth, whereas associations were weaker for average exposure over the entire pregnancy. For example, average term birth weight was 8.9 g lower (95% CI: –16.2, –1.5) in association with a 9.0°C IQR increase in Ta during the 7 days before birth; 16.6 g lower (95% CI: –27.4, –5.9) and 16.7 g lower (95% CI: –29.7, –3.7) for the 30 days and last trimester before birth, respectively (IQR increase of 9.1°C and 8.4°C); and 5.0 g lower (95% CI: –7.8, –2.3) with an IQR increase of 2.7°C in average Ta over the entire pregnancy.

The OR for low term birth weight with a 2.7°C increase in model-based Ta over the entire pregnancy was 1.04 (95% CI: 0.96, 1.13), compared with 1.07 (95% CI: 0.87, 1.27) for monitor-based Ta (Table 5). The OR for preterm birth with a 2.7°C increase in model-based Ta over the entire pregnancy was 1.04 (95% CI: 0.96, 1.13) compared with 1.02 (95% CI: 1.00, 1.05) for monitor-based Ta.

**Table 5:** Accelerated failure time model (AFT) results on the relationship between gestational age and Ta (*n *= 473,977) and logistic model results on preterm (*n *= 473,977) and low birth weight outcomes (*n *= 453,658).

Outcome and exposure time period	Predicted air temperature (°C)	Closest monitor temperature (°C)
AFT model (gestational period) β (95% CI)
Last trimester	–0.0015 (–0.0026, 0.0005)	0.0037 (0.0037, 0.0038)
Entire pregnancy	–0.0026 (–0.0028, –0.0025)	0.0089 (0.0088, 0.0090)
Preterm births (< 37 weeks) [OR (95% CI)]
Entire pregnancy	1.02 (1.00, 1.05)	1.07 (0.87, 1.27)
Low birth weight (< 2,500 g) [OR (95% CI)]
Entire pregnancy	1.04 (0.96, 1.13)	1.02 (0.45, 2.30)
All models adjusted for predicted air temperature, predicted PM_2.5_, cumulative traffic density, percent of open spaces, age of mother, chronic conditions of mother or conditions of pregnancy (lung disease, hypertension, gestational diabetes or non-gestational diabetes), parity, previous infant weighing ≥ 4,000 g and sex of infant, sine and cosine (controlling for seasonality), APNCU (as a categorical variable), mother’s race (as a categorical variable), mother’s education (as a categorical variable), and previous preterm occurrences.		

A 2.7°C increase in Ta over the entire pregnancy was associated with a 0.26% decrease in gestational age (95% CI: –0.28, –0.25%), and an 8.4°C increase in Ta over the last trimester before birth was associated with a 0.15% decrease in gestational age (95% CI: –0. 26, 0.05%) (Table 5). For monitor-based Ta, the results were significant as well, but showed an increase in gestational age: a 0.89% increase in gestational age (95% CI: 0.88, 0.90%) for full-term birth and 0.37% increase in gestational age (95% CI: 0.37, 0.38%) for the last trimester.

The association between an IQR increase in predicted Ta during the entire pregnancy and birth weight was stronger among births to mothers with residences in urban areas (< 30 km from a monitor, 8.1 g lower; 95% CI: –12.2, –4.0) compared with births to mothers residing in rural areas (> 30 km from a monitor, 4.2 g lower; 95% CI: –8.4, 0.1), though the differences were not statistically significant (interaction *p*-value = 0.26).

## Discussion

In the presented study, we estimated the associations of Ta on birth outcomes in a study of singleton births in Massachusetts counties between 2000 and 2008. Using a model enhanced with satellite remote sensing, we were able to assign exposure to all subjects with less spatial and temporal error (compared with using a closest-monitor approach), regardless of the distance between a participant’s residence and the closest Ta monitor.

We found a consistent negative association between Ta and birth weight for infants who were born full term after adjusting for other potential risk factors, such as previous and current mother’s health conditions, socioeconomic factors, and physical environment risk factors such as traffic density in surrounding grid cells. The association with Ta over the entire pregnancy was stronger in more urban areas (< 30 km from a monitor) than in more rural areas (≥ 30 km from a monitor), though the difference was not statistically significant. In contrast to the associations found with our modeled predicted Ta, associations between birth weight and Ta measured at the nearest ground monitor stations were close to the null, suggesting that predicted Ta classified exposure more accurately than monitor-based estimates. Interestingly, for the AFT analysis we found that an increase in Ta over both periods were associated with a decrease in gestational age; yet in the monitored Ta analysis, these associations were significantly associated with an increase of gestational age. These findings need to be further explored in future studies.

A key advantage of the presented study is the exposure assignment. Because our model allows us to predict temporally and spatially resolved Ta, we can assign daily Ta exposure to the entire study population, avoiding potential selection bias that would yield a nonrepresentative sample. It also captures the urban heat island effect, as shown in Figure 2. In addition, we account for small area measures of potential confounders at a 1 × 1 km spatial resolution such as individual and census measures of socioeconomic status, and medical history.

The literature on the potential impact of Ta on birth weight and its determinants is still very limited. Increased Ta may affect birth weight through direct or indirect means. The causes of preterm birth and LBW are largely unknown, but are likely to be a complex mix of genetic, behavioral, socioeconomic, and environmental factors (Strand et al. 2011). Heat stress during spells of high Ta has been suspected as a cause of premature birth, resulting in high prevalence of LBW (Basu et al. 2010). Pregnant women may be more susceptible to changes in temperature because of the extra physical and mental strain, and may be at a greater risk of heat stress because of multiple factors, such as increased fat deposition; the ratio of surface area to body mass, which decreases, reducing the capacity to lose heat by sweating; weight gain, which increases heat production; and the fetus adding to the maternal heat stress by adding its own body’s composition and its own metabolic rate (Wells and Cole 2002). Three studies have reported positive associations between preterm birth and Ta (Flouris et al. 2009; Lajinian et al. 1997; Yackerson et al. 2008), but two other studies did not report an association (Lee et al. 2008; Porter et al. 1999).

Race, ethnicity, education, and other socioeconomic status factors are often clustered spatially and can act as potential confounders since they do not vary by time but do vary by space. We use a random-effects model with a random intercept for FIPS (Federal Information Processing Standard) code while controlling for seasonality to reduce bias as well.

There are several limitations in the present study. First, the spatial resolution of the exposures was 1 × 1 km. As satellite remote sensing evolves and progresses, higher spatial resolution data should become available in the coming years, which will further reduce exposure error. Such increased resolution should enable us to more precisely estimate daily intraurban exposures and how these vary across spatial locations. Other limitations include the lack of some health and personal-level data such as maternal weight, body mass index, differences across different locations in physical activity, and pollen exposure. We also lacked data on indoor temperature exposure and information on air conditioning use in households. Finally, another limitation is the lack of information on road noise as in some recent pregnancy outcome studies (Dadvand et al. 2014; Gehring et al. 2014).

In summary, our findings suggest that higher Ta during pregnancy may be a risk factor for lower birth weight.
